# University students experience the COVID-19 induced shift to remote instruction

**DOI:** 10.1186/s41239-021-00296-5

**Published:** 2021-11-17

**Authors:** Bob Ives

**Affiliations:** College of Education and Human Development, University of Nevada, Reno, MS0299, Reno, NV 89557-0299 USA

**Keywords:** COVID-19, Online, Accessibility, Quality of instruction, University

## Abstract

The COVID-19 pandemic required an abrupt shift from face-to-face to online instruction for many students in higher education in the United States. Prior research has raised some concerns about both equitable access to online courses, and the quality of instruction in online courses compared to face-to-face courses. This survey study included a retrospective pretest approach to comparing students experiences before and after the transition to online instruction. The sample of 1731 students ranged across all available topics of study and all class standings from first-year students to doctoral students at a R1: Doctoral Universities—Very High Research Activity university according to the Carnegie classifications. Quality of instruction was addressed through the three principles of Universal Design for Learning. Students reported that most areas of quality of instruction were poorer after the transition, with having Engagement dropping by the largest effect size. However, Representation showed a small effect of improvement following the transition. Students who preferred online instruction reported less loss of instructional quality. Similarly, students eligible for disability services also reported less loss of instructional quality. Doctoral students reported significantly poorer access on multiple measures compared to all four years of undergraduate students’ standings. Results are discussed in terms of patterns, exceptions, effect sizes, and recommendations for future research.

**Supplementary Information:**

The online version contains supplementary material available at 10.1186/s41239-021-00296-5.

## Introduction

The abrupt change in higher education from face-to-face to online learning in the Spring of 2020 raised concerns about the accessibility of online instruction, as well as the quality of instruction in online learning (Lassoued et al., 2020). As of this writing, a search for “COVID” through the Educational Resources Information Center (ERIC) database yielded 835 results. About half of those did not even mention “online” instruction, and most of those that remain were not based on original empirical research. The remaining research studies were not based on data from students in higher education to address concerns about access and quality of instruction related to this transition to online instruction.

A review of studies addressing the COVID-19 pandemic and higher education found that the large majority of studies were descriptive, and that previous reviews focused primarily on institutional processes (Bond, in press). The purpose of this study was to map the content of original relevant research, rather than to synthesize their findings. More than half of the studies in this recent review focused on the experiences of undergraduates with respect to teaching and learning, and only two of those studies focused on students with disabilities, whereas the present study sought to also include postgraduate students and focus on students who are eligible for disability services.

## Accessibility

Some international studies have looked at the issue of access related to the transition to online instruction caused by the pandemic. For example, two studies of students in Turkey reported that problems with technology hindered their learning after the transition to online learning (Arici, 2020; Hebebci et al., 2020). Algerian students reported similar problems (Blizak et al., 2020), as did students in Saudi Arabia (Al-Nofaie, 2020). These studies did not included data about accessibility prior to the pandemic for comparison.

While studies of accessibility specifically related to the transition to online instruction because of the current pandemic are limited, there is more research available about accessibility issues related to online learning in general. For example, while post-secondary students prefer face-to-face learning (Sutiah et al., 2020), some concerns have been raised about accessibility to online content and resources for particular populations including students with disabilities and low income students. For example, students with visual impairments have problems with accessibility to massive open online courses (MOOCs) (Park, 2019), virtual reality applications (Lannan, 2019), and information and communication support technology (Eligi, 2017). Students who are deaf or hard of hearing also report problems with accessibility to online learning (Batanero et al., 2019; Ferreiro-Lago & Osuna-Acedo, 2017). At the same time, students with a variety of disabilities report preferring online learning (Ilgaz & Gulbahar, 2017; Kent et al., 2018), despite the preference of most students for face-to-face classes. Nevertheless, international analyses of online learning accessibility in general has found learning materials and sites wanting (Alsalem and Abu, 2018; Boateng, 2016; Carvajal et al., 2018; Massengale & Vasquez III, 2016).

Low income students, first generation students, and older students also are more likely to have problems taking advantage of online courses because of both access challenges and less experience and expertise with related technology (Buzzetto-Hollywood et al., 2018). For example, a recent study found that access to technology for low income students was exacerbated by the transition to online instruction required by the current pandemic (Kim & Padilla, 2020). Banerjee (2020) confirmed that first generation students have poorer access to technology. The digital divide between older and younger people in general has been documented, although the research on an age-based digital divide specifically in education is limited (Blažic & Blažic, 2020).

### Quality of Instruction

The learning platform company Top Hat (2020) surveyed over 3,000 college and university students in the United States and Canada about their experiences with online learning during the fall of 2020. These students reported a reduction in engagement and motivation related to remote learning. These students overwhelmingly preferred face-to-face over remote instruction, and also preferred synchronous remote instruction with live streaming and chat over asynchronous remote instruction. They also recommended a stronger emphasis on active learning and community building in online courses.

A survey of Indonesian students found that students were dissatisfied with communication with their instructors, and with the quality of knowledge transfer, after the transition to online instruction, although there were no results from before that transition for comparison (Syauqi et al., 2020). Students at a university in the United States stressed the need for good communication with their instructors after the transition (Murphy et al., 2020). Authors of another study of students in the United States concluded that students engagement was negatively impacted by the transition (Perets et al., 2020). As is common for research into student engagement, the property of engagement is not well defined in these studies (Bond et al., 2020).

Universal Design for Learning (UDL) is an evidenced-based approach to instructional design for effective and inclusive learning experiences. UDL is based on three broad principles (CAST, 2021). The Engagement principle is based on multiple means to motivate learners – the WHY of learning. Following the Presentation principle ensures that content is presented in multiple ways – the WHAT of learning. The Action & Expression principle focuses on multiple means for learners to interact with the content and express what they know – the HOW of learning.

Recent reviews and meta-analyses have confirmed that UDL is effective in traditional face-to-face classes (Al-Azawei et al., 2016; Capp, 2017). However, evidence for the effectiveness of UDL in online education is more limited. Scholars have recommended the application of UDL to online instruction (Catalano, 2014; Pittman & Heiselt, 2014). However, instructors have expressed concerns about implementing UDL in online courses because of their discomfort with technology, pedagogical competencies, available time, and resistance to change (Singleton et al., 2019).

There is some research to support the incorporating UDL guidelines into online courses to improve the quality of instruction. For example, students reported better communication about expectations and other course information after UDL was applied to the redesign of an online undergraduate course (Rao & Tanners, 2011). When instructors applied the principle of Action & Expression to a final course project, students reported positive engagement and learning from the project (Boothe et al., 2020). Similar results were reported from students when entire graduate level courses were designed to incorporate UDL principles (Scott et al., 2015). Implementation of UDL in online undergraduate classes was also a predictor of student acceptance of online learning (Al-Azawei et al., 2017).

The COVID-19 pandemic prompted an abrupt shift from face-to-face to remote instruction in universities. However, prior research has raised concerns about the quality of instruction in online courses, and well as equity and accessibility issues for online courses. At the same time, relevant research based on student responses is limited, and often does not include comparison data about experiences before the transition to online instruction. In addition, as the overall use of online instruction continues to increase, perhaps with some additional impetus from experiences with the COVID-19 pandemic, the implications for research on these issues are broad, and have long-term importance. For these reasons, I addressed the following three research questions:

What changes in quality of instruction did university students experience related to the transition to remote instruction due to the pandemic?

Were quality of instruction experiences different for university students eligible for disability services?

Was access to instruction and course materials different across specialties, classes, and whether or not students preferred online instruction over face-to-face instruction?

Was access to instruction and course materials different for university students eligible for disability services?

## Methods

The project proposal was reviewed and approved by an ethics review process that is mandated by federal law in the United States, and the project was carried out without any deviations from the original proposal (Project Number: 1646025–1. Data were collected through an anonymous, and voluntary, online survey. Some items were based on a retrospective pretest–posttest design to identify changes in perceived experiences. Although this was an original survey, most of the items were based on previous research, as described below. Based on university records, all students enrolled during the spring 2020 semester were invited by email to participate in the study. They were given three weeks to complete the survey, and a reminder was sent to the same email list halfway through the three-week window. A total of 1,731 students responded from a distribution list of 19,752.

### Context and participants

Data were collected from 1731 students at a Very High Research Activity university in the western part of the United States. Prior to the COVID-19 pandemic, the large majority of courses were taught in face-to-face classrooms. In March of 2020, all classes shifted to a totally online format, and that mandated online format continued through the early summer of 2021, with plans to return to face-to-face instruction in the fall of 2021. Students were invited to participate in the study on September 24, 2020, by email, with two follow-up reminders during the following four weeks.

### Retrospective pretest

Given the circumstances surrounding the shift from face-to-face to remote instruction in higher education, related to the COVID-19 pandemic, randomized controlled trials (RCTs) would not be practical for examining the effects of this shift on student experiences. Under theses circumstances, a retrospective pretest design may be more appropriate (Pelfrey et al., 2009). The retrospective pretest design “involves asking participants at the time of the posttest to retrospectively respond to questionnaire items thinking back to a specified pretest period. In effect, participants rate each item twice within a single sitting (“then” and “now”) to measure self-perceptions of change” (Little et al., 2020, p. 175). Retrospective pretest designs have been used in the field of education to examine the effectiveness of academic instruction (Coulter, 2012), professional development (Sullivan & Haley, 2009), and teacher efficacy beliefs (Cantrell, 2003).

Further, response shift bias poses a threat to internal validity for RCTs (Howard & Dailey, 1979; Howard et al., 1979). Response shift bias occurs when the standards participants use for responding to self-report measures changes over time in repeated measures studies. In the case of the pandemic-induced shift to online instruction, responses about online instruction may be influenced by the experience of shifting from face-to-face to remote instruction, so that responses before the shift are based on different expectations from responses after the shift. In addition, RCTs, by definition, cannot be applied in situations where the researcher does not have control over the predictor variable and participants cannot be randomly assigned to different conditions. For these reasons, a retrospective pretest design offers a useful approach for comparing student experiences before and after the shift to online instruction.

### Instrument

The instrument included two demographic items based on an earlier internal survey conducted by the university. These two items asked for academic standing (class), and major. The survey also included an item asking “How many university-level online courses had you completed before the Spring of 2020?” (Wang, 2014).

Four items asked about the quality of access students had to the course content. These items asked about the reliability of their Internet service, access to communication software (e.g. Zoom), reliability of devices such as computers and smart phones, and quality of experiences with online replacements for face-to-face collaboration (e.g. digital breakout rooms, white boards, discussion groups, etc.) (Gladhart, 2010; Murphy et al., 2019).

One dichotomous item asked if participants preferred online or face-to-face learning (Erickson & Larwin, 2016; Ilgaz & Gulbahar, 2017; Kent et al., 2018). Another dichotomous item asked if participants were eligible for disability services at the university. This item was based on the previous internal survey.

Four items asked about the frequency and helpfulness of communications with instructors before and after the pandemic-induced transition to online learning (Wang, 2014). Six items asked about instructors’ implementation of the three principles of Universal Design for Learning before and after the transition to online learning (Rao et al., 2015; Rao & Tanners, 2011; Singleton et al., 2019; Westine et al., 2019). The instrument is included in Additional file 1: Appendix S1 of this manuscript.

### Data analysis

- What changes in quality of instruction did university students experience related to the transition to remote instruction due to the pandemic?
  The results from a series of paired sample t-tests addressed this research question (Sagarin et al., 2014). For each of these tests, the mean of students’ reported experiences before the transition to online instruction, were compared to the mean of students’ reported experiences after the transition to online instruction.
- Were quality of instruction experiences different for university students eligible for disability services?
  Using one-way ANOVAs, I tested the differences in gain score means for students eligible for disability services versus students not eligible for disability services. Because some comparisons failed a homogeneity of variance test, results for the Welch statistic are reported (Sagarin et al., 2014), to adjust for problems with homogeneity of variance.
- Was access to instruction and course materials different across specialties, classes, and whether or not students preferred online instruction over face-to-face instruction?
  An omnibus ANOVA was run on responses for each of the four items related to access to determine if there were any significant differences across these specialties, with planned Tukey tests to identify pairwise significant differences, if any. A parallel analysis was planned and conducted to compare mean responses to the same four items across seven groups of students based on their academic standing – first year, sophomore, junior, senior, masters, doctoral, and graduate students who are not in a degree program. Using one-way ANOVAs, I compared the accessibility mean scores across students who preferred online instruction versus those who preferred face-to face instruction. Because some of these comparisons failed a test of homogeneity of variance, Welch statistics are reported.
- Was access to instruction and course materials different for university students eligible for disability services?

ANOVA was used to compare the scores from the four access items for the eligible students, with the scores on those items for the rest of the participants.

Given that a large number of statistical tests were run for this study, the results are vulnerable to Type 1 error inflation (Sagarin et al., 2014). In addition, most of the sample sizes are large, providing enough statistical power to detect quite small effects. For these reasons, results will primarily be discussed in terms of overall patterns, exceptions to patterns, and effect sizes. When interpreting the results, it is also important to recognize that the participants are reporting their own experiences, which are not confirmed by independent measures. In addition, the study involved no manipulation of intervention or randomization of participants into groups. For both of these reasons, causal inferences, and recommendations for interventions must be speculative before confirming research results are available.

## Results

### What changes in quality of instruction did university students experience related to the transition to remote instruction due to the pandemic?

Table 1 reports the results from a series of t-tests checking for significant differences between retrospective pretest response means and posttest response means for each of the three UDL principles as well as the two issues of instructor communication. The table includes the number of students who responded to both items (N), the test statistic (*t*), the probability of a Type I error if the null hypothesis is rejected (*p*), and a standardized mean difference effect size (g*). A standardized mean difference is independent of statistical significance, making it insensitive to sample sizes, and generalizable across different analyses and studies (Ives, 2003). However, Cohen’s *d* and Hedges *g* are both susceptible to small sample bias. The effect size measure I used was Hedges *g* with a correction for this small sample bias (Durlak, 2009; Hedges & Olkin, 1985). Although our sample size would not be considered small, I adopted this effect size measure as a matter of good practice and consistency.

**Table 1:** Significant changes in reported quality of instruction before and after the shift to remote learning

***Item***	***N***	***M (SD)***	***t***	***p***	g*
*Poorer After the Transition*					
Frequency of Communication with the Instructor	1210	Before 2.14 (1.009) After 3.02 (1.2720	26.069	< .001	.99
Helpfulness of Communication with the Instructor	1208	Before 2.10 (1.047) After 2.73 (1.252)	22.498	< .001	.74
Action & Expression (Assessment in Different Ways)	1207	Before 1.90 (1.013) After 2.17 (.970)	15.332	< .001	.45
Engagement	1202	Before 2.10 (.727) After 1.35 (.616)	41.920	< .001	1.68
*Better After the Transition*					
Representation (Presenting Content in Different Ways)	1206	Before 2.10 (.857) After 1.78 (.864)	.8700	< .001	.30

All five of these comparisons yielded statistically significant differences between experiences before and after the shift to online instruction induced by the pandemic. In addition, these results show that these tests have adequate statistical power to detect small effects (Cohen, 1988). Four of the measures of quality of instruction became poorer after the switch to online instruction. Two of these had large effect sizes (frequency of communication, and engagement), one had a medium effect size (helpfulness of communication), and one had a small effect size (action & expressions). In addition, students reported that representation improved after the transition to on line instruction, with a small effect size.

Two hundred and twenty-eight of the participants reported preferring online classes over face-to-face instruction, while 1,125 reported preferring face-to-face instruction. For all participants, quality of instruction scores before the online shift were subtracted from quality of instruction scores after the shift to create gain scores for each participant, for each of the five measurers of quality of instruction. Using one-way analysis of variance (ANOVAs), I compared the quality of instruction gain scores across these two groups. Because some of these comparisons failed a test of homogeneity of variance, Welch statistics are reported. Results are reported in Table 2. In every case, students who preferred face-to-face instruction also reported significantly poorer experiences with quality of instruction than students who preferred online instruction. Three of the effect sizes are large, while the other two are small.

**Table 2:** Comparison of gains scores between students who preferred online instruction versus those who preferred face-to-face (f2f) instruction for quality of instruction before and after the shift to remote learning

*Item*	*N*	*M (SD)*	*Welch*	*p*	g*
Frequency of Communication with the Instructor	Online 214 f2f 990	− .0607 (1.10952) − 1.2111 (1.29901)	177.463	< .001	1.00
Helpfulness of Communication with the Instructor	Online 213 f2f 989	.0282 (.90552) − .9858 (1.23700)	190.526	< .001	.95
Action & Expression	Online 212 f2f 989	− .0613 (.77338) − .4692 (.91563)	45.335	< .001	.40
Engagement	Online 211 f2f 986	.0379 (.93528) 1.1988 (.72731)	287.817	< .001	1.40
Representation	Online 213 f2f 987	.6291 (1.04993) .2057 (1.08524)	28.153	< .001	.48

### Were quality of instruction experiences different for university students eligible for disability services?

One hundred and forty-seven of the participants reported being eligible for disability services. Descriptive statistics for both eligible students and other students are reported in Table 3. Negative mean gain scores indicate a reduction in instructional quality. Consistent with the findings for the first research question, both groups reported positive gain scores for the Representation gain scores, indicating an improvement in Representation following the transition to online instruction, and students eligible for disability services reported a greater improvement in Representation. Both groups reported poorer quality for all four of the other items related to quality of instruction, but in each case, students eligible for disability services reported less of a drop in instructional quality. Overall, the impact of the move to online instruction seemed to have a less negative effect for students eligible for disability services.

**Table 3:** Descriptive statistics for quality of instruction items for students eligible for disability services and other students

Item	Eligibility	N	M	SD
Frequency of communication	Eligible	135	− .8815	1.50646
	Other	1070	− 1.0252	1.31918
Helpfulness of communication	Eligible	134	− .6343	1.24780
	Other	1069	− .8297	1.24560
Representation	Eligible	134	.3209	1.02305
	Other	1067	.2680	1.10318
Action & expression	Eligible	134	− .2761	.82619
	Other	1068	− .4157	.91505
Engagement	Eligible	133	− .7519	.99549
	Other	1065	− 1.0254	.86620

**Table 4:** Differences in quality of instruction gain scores between students eligible for disability services and other students

Item	Welch Statistic	p	g*
Frequency of Communication	1.121	.291	.11
Helpfulness of Communication	2.922	.089	.16
Representation	.312	.577	.05
Action and expression	3.316	.070	.15
Engagement	9.170	.003	.31

**Table 5:** Means (standard deviations) across specialties for the four access items

Specialty	N	Internet service	Communication software	Devices	Collaboration tools
Agri, Biotech, Nat Res	108	2.40 (.995)	2.36 (1.045)	2.17 (.922)	3.30 (1.078)
Business	182	2.23 (.964)	2.20 (.930)	2.05 (.968)	3.48 (1.183)
Education	114	2.26 (.987)	2.14 (.822)	2.02 (.872)	2.83 (1.056)
Engineering	181	2.28 (.984)	2.25 (.960)	2.14 (1.045)	3.56 (1.078)
Liberal Arts	184	2.40 (.997)	2.29 (.965)	2.20 (1.053)	3.27 (1.190)
Science	281	2.33 (.990)	2.21 (.941)	2.15 (.951)	3.30 (1.099)
Nursing	39	2.33 (.869)	2.28 (.686)	2.18 (.756)	3.21 (1.056)
Comm health sciences	125	2.41 (.960	2.35 (.915)	2.24 (.902)	3.38 (1.169)
Journalism	25	2.60 (.764)	2.56 (.821)	2.68 (.988)	3.64 (1.150)
Medicine	20	2.40 (1.046)	2.40 (.883)	2.55 (.945)	3.45 (1.234)
Social work	35	2.29 (.957)	2.23 (.646)	2.31 (.963)	2.97 (1.031)
Undeclared	14	2.29 (1.069)	2.21 (1.188)	2.14 (1.027)	3.50 (1.137)

**Table 6:** Means (standard deviations) across class standing for the four access items

Standing	N	Internet service	Communication software	Devices	Collaboration tools
First year	219	2.37 (.998)	2.26 (.903)	2.17 (.989)	3.37 (1.104)
Sophomore	285	2.42 (.875)	2.40 (.942)	2.19 (.934)	3.64 (1.118)
Junior	337	2.38 (.956)	2.35 (.930)	2.24 (.969)	3.33 (1.117)
Senior	256	2.39 (1.054)	2.34 (.938)	2.22 (1.031)	3.37 (1.081)
Masters	102	2.08 (.992)	1.91 (.873)	2.01 (.939)	2.98 (1.160)
Doctoral	88	1.95 (.982)	1.69 (.701)	1.81 (.800)	2.52 (.919)
Graduate (non-degree)	23	2.04 (.767)	2.04 (.825)	1.87 (.869)	2.74 (1.137)

**Table 7:** Significance (effect sizes) for significant differences across class standing for the four access items

	Sophomore	Masters	Doctoral
*Internet service*			
First year			.012 (.43)
Sophomore		.039 (.35)	.002 (.48)
Junior			.005 (.44)
Senior			.005 (.45)
*Communication software*			
First year		.023 (.38)	< .001 (.61)
Sophomore		.000 (.53)	< .001 (.76)
Junior		.001 (.47)	< .001 (.71)
Senior		.001 (.46)	< .001 (.70)
*Devices*			
First Year			.042 (.37)
Sophomore			.020 (.39)
Junior			.003 (.44)
Senior			.009 (.42)
*Collaboration tools*			
First Year			< .001 (.75)
Sophomore		< .001 (.58)	< .001 (.99)
Junior	.009 (.27)		< .001 (.80)
Senior		.049 (.34)	< .001 (.84)
Graduate (non-degree)	.003 (.79)

**Table 8:** Comparison of gains scores between students who preferred online instruction versus those who preferred face-to-face (f2f) instruction for accessibility before and after the shift to remote learning

*Item*	*N*	*M (SD)*	*Welch*	*p*	g*
Internet Service	220 1084	2.00 (.963) 2.40 (.966)	32.314	< .001	.41
Communication Software	218 1082	1.87 (.801) 2.34 (.935)	59.939	< .001	.54
Devices	220 1085	1.83 (.874) 2.23 (.974)	37.018	< .001	.43
Collaboration Tools	211 1058	2.36 (1.070) 2.52 (1.048)	208.468	< .001	1.09

The results for the comparisons between groups are reported in Table 4. Although the effect of the move to online instruction was less negative for students eligible for disability services for all five measures of instructional quality, only the Engagement comparison reached a conventional level of statistical significance. This was also the only comparison for which the effect size rose to the level of a small effect, indicating that students eligible for disability services had a significantly smaller reduction in engagement in their classes that students who were not eligible.

### Was access to instruction and course materials different across specialties or classes or preferences for online or face-to face instruction?

Student specialties were identified by the university college or school that housed their primary field of study, and categorized based on the international standards established by the United Nations Educational, Scientific and Cultural Organization (UNESCO Institute for Statistics. (2015). *International Standard of Classification: Fields of Education and Training 2013 (ISCED-F 2013)—Detailed Field Descriptions*. Retrieved from Montreal, 2013). Although students studying journalism reported the best experiences with all four items, and students studying education reported the poorest experience for three of the four items, there were no statistically significant differences between the means of any of the 11 units or students who were undeclared. Means and standard deviations for these comparisons are reported on Table 5.

For the analysis across classes, all four of the omnibus ANOVAs were significant. Tukey tests identified several significantly different pairs of means for each of the four access items – a total of 25 significant pairwise comparisons. Across all four items, doctoral students reported poorer experiences with access than each of the four undergraduate classes, accounting for 16 of the significant mean differences. Effect sizes for these differences spanned the range from medium to large. Seven of the remaining significant differences were between masters students and some of the undergraduate classes. The effect sizes for these differences were almost all in the small range. Only one of the significant comparisons involved comparing undergraduates to undergraduates, and one involved comparing sophomores to graduate students who were not in a degree program. Table 6 reports means and standard deviations for the four access items across class standing. Table 7 reports *p*-values and effect size measures for the significant pairwise comparisons.

Comparisons of accessibility between students who preferred face-to-face instruction and those who did not are reported in Table 8. In every case, students who preferred face-to-face instruction also reported significantly poorer experiences with accessibility than students who preferred online instruction. Effect sizes ranged from small to large.

### Was access to instruction and course materials different for university students eligible for disability services?

The sample of participants for this study included 153 students who reported being eligible for disability services. The eligible students reported better access for three of the four items, and poorer access for one of them. However, none of the mean differences between the two groups approached significance (all *p*-values were > 0.18), suggesting that the access experiences of the two groups were similar.

## Discussion and conclusion

### Quality of instruction

Based on prior research, a drop in reported quality of instruction would be expected after the transition to online instruction. At the same time, perceived quality of online instruction is related to how accepting students are of online instruction (Larmuseau, 2019). Our own results found this to be true for both the frequency and helpfulness of instructor communication. This result is consistent with prior work showing that instructor availability is a predictor of student perceptions of quality of instruction in online classes (Slaydon et al., 2020). These results suggest that instructors could improve the perceived quality of their online instruction by enhancing their availability and communication with students.

Also related to quality of instruction, students in this study reported that two principles of Universal Design for Learning (UDL), Engagement and Action & Expression, were significantly poorer after the transition to online instruction. In fact, the drop in Engagement was the largest of all the effect sizes for the five measures of quality of instruction. Synchronous online activities are related to improved engagement in online classes (Weiler, 2012), and prior research has shown that some design elements for online activities are more effective at enhancing student engagement than others (Cundell & Sheepy, 2018).

Students reported that the Representation element of UDL actually improved after the transition to online learning. This result held across all participants, as well as the separate subgroups of participants eligible or not eligible for disability services. One hypothesis to explain this finding is that the constraints of online instruction may require that instructors to be more creative about their presentation of content than in face-to-face classrooms. This hypothesis should be addressed in future research.

Changes in perceived quality of instruction may be attributable to changes in actual quality of instruction. However, other variables may also help to account for these changes. For example, a general perception that online instruction is of poorer quality may introduce a bias in perceptions relative to face-to-face instruction. In this context, students who preferred online over face-to-face instruction reported significantly more positive experiences about quality of online instruction, compared to face-to-face instruction, than did students who preferred face-to-face instruction.

These results regarding the quality of instruction suggest that the impact of the shift to online instruction related to the COVID-19 did not result in a uniform overall reduction in quality of instruction. Instead, the impact may be more complex, with some elements of instructional quality actually benefitting from the shift to online instruction. Most notably, the Representation principle of UDL improved after the move to online instruction. This complexity warrants further research to understand more clearly where quality of instruction would most benefit from improved resources.

Students who were eligible for disability services reported a significantly smaller drop in engagement after the online transition, than other students reported. Students eligible for disability services also reported less negative changes for the other four measures of quality of instruction, but these mean differences were not statistically significant. A plausible hypothesis to be tested by future research would be that contact with campus offices providing disability services may have helped to support the engagement of eligible students. The other four measures of quality of instruction are more directly related to what is happening in the course, and may not benefit from the work of the support staff providing disability services. The results of this study indicate that the shift to online instruction may have differentially impacted some students more than others. While this possibility warrants further investigation, these differential impacts may justify more differentiated interventions for student support.

### Accessibility

There were no statistically significant differences between means across colleges or schools within the university for any of the four measures of accessibility. In addition, there were no significant differences on the accessibility measures between students eligible for disability services, and those who were not.

Across students with different class standing, the results were more varied. The most striking pattern was that doctoral students reported more difficulty on all four of the measures of accessibility than any of the four undergraduate groups. The effect sizes were mostly small for Internet access and reliability of devices, medium for communication software, and large for collaboration tools. This pattern might suggest that doctoral students were getting less support for accessibility, but it is not clear why that might be. Some evidence suggests that doctoral students, at least in the United Kingdom, found the pandemic lockdown interfered with their ability to pursue their research activities, while they also had no assurance of extensions or other considerations from their universities. (Byrom, 2020). Another hypothesis is that doctoral students are typically older than undergraduates, and doctoral students, at least in some fields, may be less facile with digital technologies. This is a troubling finding that warrants further investigation. Masters students also reported significantly greater difficulty with communication software than each of the four undergraduate classes, and these effect sizes were in the small range. For these results, the shift to online instruction has differentially impacted accessibility issues for some students more than others. This research, and systematic replications, can guide decisions about student support.

Not surprisingly, students who preferred online instruction reported significantly less difficulty with accessibility for all four measures. Students who preferred online instruction also reported having taken significantly more online courses than other students prior to the transition to online instruction (Welch = 292.581, *p* = 0.009, g* = 0.21). Three of those results yielded small effect sizes, while the result for collaboration tools yielded a large effect size. Perhaps collaboration tools are particularly vulnerable to the transition to online instruction. This pattern is reflected in the results for doctoral students in the previous paragraph.

The circumstances of the COVID-19 pandemic should be taken into consideration when interpreting these results. First, the shift to online instruction was mandated rather than voluntary. Second, the initial transition in the spring of 2020 occurred while classes were already in progress. That means students made the initial transition to online instruction within the same classes they were already taking face-to-face. Third, during the pandemic, students faced additional emotional challenges that may have influenced their experiences with the transition. It is not clear what these results might imply for situations where students have voluntarily chosen online versus face-to-face instruction when not facing a significant crisis like the COVID-19 pandemic.

In most cases, these results reflect perceived losses in both quality of instruction and accessibility to online resources and content following the transition to online instruction impelled by the COVID-19 pandemic. While acknowledging the risks of overgeneralizing and overinterpreting these results, they do support a recommendation that institutions of higher education focus on helping instructors improve quality of online instruction and access, particularly in the areas of student engagement, instructor communication, and use of collaboration tools. In addition, more focus on supporting quality of instruction and access for doctoral students is supported by these data. At the same time, there are some encouraging results as well. The representation principle of Universal Design for Learning reportedly improved after the shift to online instruction. In addition, students eligible for disability services reported less of a loss in quality of instruction and access than other students reported. Given that the use of online instruction in higher education was increasing before the COVID-19 pandemic, and maybe given further impetus from this pandemic, the implications for research on these issues are broad, and have long-term importance. The results of this study justify conducting similar studies, not just for systematic replication, but because the results can inform policy and practice in higher education online instruction.

## Supplementary Information

**Additional file 1: Appendix S1.**

## Data Availability

Data and materials are available from the author. The instrument is attached as an Additional file 1: Appendix S1.

## Abbreviations

ANOVA: Analysis of variance
ERIC: Educational Resources Information Center
MOOCs: Massive open online courses
RCTs: Randomized controlled trials
UDL: Universal Design for Learning
