# Supplementary material for: University students experience the COVID-19 induced shift to remote instruction
Source: Int J Educ Technol High Educ. 2021 Nov 17;18(1):59. doi: 10.1186/s41239-021-00296-5 (PMC8594957; doi:10.1186/s41239-021-00296-5)
Supplement: Supplementary file 1 — Additional file 1: Appendix S1. [file 41239_2021_296_MOESM1_ESM.docx]

**Appendix**

Q1 What was your academic standing last Spring semester, 2020?

- First Year Student (1)
- Sophomore (2)
- Junior (3)
- Senior (4)
- Masters Degree Program (5)
- Doctoral Degree Program (6)
- Graduate Special (7)

Q2 What unit is your primary major in?

- CABNR (1)
- Business (2)
- Education (3)
- Engineering (4)
- Liberal Arts (5)
- Science (6)
- Orvis School of Nursing (7)
- Community Health Sciences (8)
- Journalism (9)
- Medicine (10)
- Social Work (11)
- Undeclared (12)

Q4 How many university-level online courses had you completed **before** the Spring of 2020? (number entry)

________________________________________________________________

Q5 What experiences did you have in your Kindergarten-12 education that helped prepare you for online classes?

________________________________________________________________

Q7 How reliable was your Internet service during the Spring 2020 semester?

- Excellent (1)
- Good (2)
- Average (3)
- Poor (4)
- Terrible (5)
- N/A (6)

Q8 How reliable was your access to communication software (e.g. Zoom, Teams, etc.) during the Spring 2020 semester?

- Excellent (1)
- Good (2)
- Average (3)
- Poor (4)
- Terrible (5)
- N/A (6)

Q9 How reliable were your computers and other devices for accessing the Internet during the Spring 2020 semester?

- Excellent (1)
- Good (2)
- Average (3)
- Poor (4)
- Terrible (5)
- N/A (6)

Q10 Rate your experience with digital replacements for face-to-face collaboration tools (e.g. digital break out rooms, whiteboards, discussions, collaborations, etc.) during the Spring 2020 semester.

- Excellent (1)
- Good (2)
- Average (3)
- Poor (4)
- Terrible (5)
- N/A (6)

Q13 What challenges did you face accessing online classes during the Spring 2020 semester?

________________________________________________________________

Q11 Do you prefer online learning or face-to-face learning?

- online learning (1)
- face-to-face learning (2)

Q12 Why do you prefer this mode of learning?

________________________________________________________________

Q15 CHOOSE ONE COURSE THAT YOU COMPLETED IN THE SPRING OF 2020 THAT TRANSITIONED FROM FACE-TO-FACE TO ONLINE INSTRUCTION. For that one course, please answer ALL of the remaining questions:

Q66 UNR students were given the opportunity to change from letter grades to S/U during the spring semester, 2020. For the course you selected, what was the scale for your final grade?

- Letter grade (1)
- changed from letter grade to S/U (2)
- S/U from the beginning of the semester (3)

Q6 If you received a letter grade (not S/U) for this course, how do you think the transition to online instruction in Spring 2020 may have affected your grade in the course?

- the transition probably made my grade higher (1)
- the transition probably made no difference in my grade (2)
- the transition probably made my grade lower (3)
- N/A, took the course S/U (4)

Q14 Are you eligible for an accommodation for technical or teaching-related accessibility? *(An accommodation is a means or method designed to assist users with disabilities.)*

- Yes (1)
- No (2)

Skip To: Q40 If Are you eligible for an accommodation for technical or teaching-related accessibility? (An accomm... = No

Q15 How was your access to**Extra Time on Tests** BEFORE the transition to online learning?

- Excellent (1)
- Good (2)
- Average (3)
- Poor (4)
- Terrible (5)
- No experience (6)

Q16 How was your access to Extra Time on Tests AFTER the transition to online learning?

- Excellent (1)
- Good (2)
- Average (3)
- Poor (4)
- Terrible (5)
- No experience (6)

Q20 How was your access to**Speech Recognition Software for Tests** BEFORE the transition to online learning?

- Excellent (1)
- Good (2)
- Average (3)
- Poor (4)
- Terrible (5)
- No experience (6)

Q17 How was your access to**Speech Recognition Software for Tests** BEFORE the transition to online learning?

- Excellent (1)
- Good (2)
- Average (3)
- Poor (4)
- Terrible (5)
- No experience (6)

Q18 How was your access to **Quiet/Private Testing Environment for Tests** BEFORE the transition to online learning?

- Excellent (1)
- Good (2)
- Average (3)
- Poor (4)
- Terrible (5)
- Click to write Choice 6 (6)

Q19 How was your access to **Quiet/Private Testing Environment for Tests** AFTER the transition to online learning?

- Excellent (1)
- Good (2)
- Average (3)
- Poor (4)
- Terrible (5)
- No experience (6)

Q21 How was your access to **Word Processors for Essay Exams** BEFORE the transition to online learning?

- Excellent (1)
- Good (2)
- Average (3)
- Poor (4)
- Terrible (5)
- No experience (6)

Q22 How was your access to **Word Processors for Essay Exams** AFTER the transition to online learning?

- Excellent (1)
- Good (2)
- Average (3)
- Poor (4)
- Terrible (5)
- No experience (6)

Q23 How was your access to **Enlarged Print for Tests** BEFORE the transition to online learning?

- Excellent (1)
- Good (2)
- Average (3)
- Poor (4)
- Terrible (5)
- No experience (6)

Q24 How was your access to **ASL Interpreters** AFTER the transition to online learning?

- Excellent (1)
- Good (2)
- Average (3)
- Poor (4)
- Terrible (5)
- No experience (6)

Q25 How was your access to **Readers for Tests** BEFORE the transition to online learning?

- Excellent (1)
- Good (2)
- Average (3)
- Poor (4)
- Terrible (5)
- No experience (6)

Q26 How was your access to **Readers for Tests** AFTER the transition to online learning?

- Excellent (1)
- Good (2)
- Average (3)
- Poor (4)
- Terrible (5)
- No experience (6)

Q27 How was your access to **Scribes for Tests** BEFORE the transition to online learning?

- Excellent (1)
- Good (2)
- Average (3)
- Poor (4)
- Terrible (5)
- No experience (6)

Q28 How was your access to **Scribes for Tests** AFTER the transition to online learning?

- Excellent (1)
- Good (2)
- Average (3)
- Poor (4)
- Terrible (5)
- No experience (6)

Q29 How was your access to **Text-to-Speech for Tests** BEFORE the transition to online learning?

- Excellent (1)
- Good (2)
- Average (3)
- Poor (4)
- Terrible (5)
- No experience (6)

Q30 How was your access to **Text-to-Speech for Tests** AFTER the transition to online learning?

- Excellent (1)
- Good (2)
- Average (3)
- Poor (4)
- Terrible (5)
- No experience (6)

Q31 How was your access to **Oral Examinations** BEFORE the transition to online learning?

- Excellent (1)
- Good (2)
- Average (3)
- Poor (4)
- Terrible (5)
- No experience (6)

Q32 How was your access to **Oral Examinations** AFTER the transition to online learning?

- Excellent (1)
- Good (2)
- Average (3)
- Poor (4)
- Terrible (5)
- No experience (6)

Q33 How was your access to **E-Text for Tests** BEFORE the transition to online learning?

- Excellent (1)
- Good (2)
- Average (3)
- Poor (4)
- Terrible (5)
- No experience (6)

Q34 How was your access to **E-Text for Tests** AFTER the transition to online learning?

- Excellent (1)
- Good (2)
- Average (3)
- Poor (4)
- Terrible (5)
- No experience (6)

Q35 How was your access to **Audio Files for Tests** BEFORE the transition to online learning?

- Excellent (1)
- Good (2)
- Average (3)
- Poor (4)
- Terrible (5)
- No experience (6)

Q36 How was your access to **Audio Files for Tests** AFTER the transition to online learning?

- Excellent (1)
- Good (2)
- Average (3)
- Poor (4)
- Terrible (5)
- No experience (6)

Q37 How was your access to **Electronic Textbooks**BEFORE the transition to online learning?

- Excellent (1)
- Good (2)
- Average (3)
- Poor (4)
- Terrible (5)
- No experience (6)

Q38 How was your access to **Electronic Textbooks** AFTER the transition to online learning?

- Excellent (1)
- Good (2)
- Average (3)
- Poor (4)
- Terrible (5)
- No experience (6)

Q52 How was your access to **DRC Exam Processing**BEFORE the transition to online learning?

- Excellent (1)
- Good (2)
- Average (3)
- Poor (4)
- Terrible (5)
- No experience (6)

Q53 How was your access to **DRC Exam Processing** AFTER the transition to online learning?

- Excellent (1)
- Good (2)
- Average (3)
- Poor (4)
- Terrible (5)
- No experience (6)

Q54 How was your access to **FM Systems**BEFORE the transition to online learning?

- Excellent (1)
- Good (2)
- Average (3)
- Poor (4)
- Terrible (5)
- No experience (6)

Q55 How was your access to **FM Systems** AFTER the transition to online learning?

- Excellent (1)
- Good (2)
- Average (3)
- Poor (4)
- Terrible (5)
- No experience (6)

Q56 How was your access to **Roger Pens**BEFORE the transition to online learning?

- Excellent (1)
- Good (2)
- Average (3)
- Poor (4)
- Terrible (5)
- No experience (6)

Q57 How was your access to **Roger Pens** AFTER the transition to online learning?

- Excellent (1)
- Good (2)
- Average (3)
- Poor (4)
- Terrible (5)
- No experience (6)

Q58 How was your access to **Video Captioning**BEFORE the transition to online learning?

- Excellent (1)
- Good (2)
- Average (3)
- Poor (4)
- Terrible (5)
- No experience (6)

Q59 How was your access to **Video Captioning** AFTER the transition to online learning?

- Excellent (1)
- Good (2)
- Average (3)
- Poor (4)
- Terrible (5)
- No experience (6)

Q60 How was your access to **TypeWell Transcription**BEFORE the transition to online learning?

- Excellent (1)
- Good (2)
- Average (3)
- Poor (4)
- Terrible (5)
- No experience (6)

Q61 How was your access to **TypeWell Transcription** AFTER the transition to online learning?

- Excellent (1)
- Good (2)
- Average (3)
- Poor (4)
- Terrible (5)
- No experience (6)

Q62 How was your access to **Notetaking**BEFORE the transition to online learning?

- Excellent (1)
- Good (2)
- Average (3)
- Poor (4)
- Terrible (5)
- No experience (6)

Q63 How was your access to **Notetaking** AFTER the transition to online learning?

- Excellent (1)
- Good (2)
- Average (3)
- Poor (4)
- Terrible (5)
- No experience (6)

Q64 How was your access to **Audio Recording of Lectures**BEFORE the transition to online learning?

- Excellent (1)
- Good (2)
- Average (3)
- Poor (4)
- Terrible (5)
- No experience (6)

Q65 How was your access to **Audio Recording of Lectures** AFTER the transition to online learning?

- Excellent (1)
- Good (2)
- Average (3)
- Poor (4)
- Terrible (5)
- No experience (6)

Q40 Rate the frequency of communication with the instructor BEFORE the transition to online learning.

- Excellent (1)
- Good (2)
- Average (3)
- Poor (4)
- Terrible (5)

Q42 Rate the frequency of communication with the instructor AFTER the transition to online learning.

- Excellent (1)
- Good (2)
- Average (3)
- Poor (4)
- Terrible (5)

Q43 Rate the helpfulness of communication with the instructor BEFORE the transition to online learning.

- Excellent (1)
- Good (2)
- Average (3)
- Poor (4)
- Terrible (5)

Q44 Rate the helpfulness of communication with the instructor AFTER the transition to online learning.

- Excellent (1)
- Good (2)
- Average (3)
- Poor (4)
- Terrible (5)

Q45 Did the instructor make course content available through multiple approaches (e.g. reading, lecture, videos, graphics, audio files, projects, etc.) BEFORE the transition to online learning.

- Often (1)
- Sometimes (2)
- Rarely (3)
- Never (4)

Q46 Did the instructor make course content available through multiple approaches (e.g. reading, lecture, videos, graphics, audio files, projects, etc.) AFTER the transition to online learning.

- Often (1)
- Sometimes (2)
- Rarely (3)
- Never (4)

Q47 Did the instructor assess your learning through multiple approaches? (tests and quizzes, group and individual projects, presentations, etc.) BEFORE the transition to online learning?

- Often (1)
- Sometimes (2)
- Rarely (3)
- Never (4)

Q48 Did the instructor assess your learning through multiple approaches? (tests and quizzes, group and individual projects, presentations, etc.) AFTER the transition to online learning?

- Often (1)
- Sometimes (2)
- Rarely (3)
- Never (4)

Q49 How engaged did you feel in this course BEFORE the transition to online learning?

- 0 (0)
- 1 (1)
- 2 (2)
- 3 (3)
- 4 (4)
- 5 (5)
- 6 (6)
- 7 (7)
- 8 (8)
- 9 (9)
- 10 (10)

Q50 How engaged did you feel in this course AFTER the transition to online learning?

- 0 (0)
- 1 (1)
- 2 (2)
- 3 (3)
- 4 (4)
- 5 (5)
- 6 (6)
- 7 (7)
- 8 (8)
- 9 (9)
- 10 (10)

Q51 After the transition to online learning, did the class continue to meet as a group online?

- Yes, at the scheduled class times (1)
- Yes, but less often than the scheduled class times (2)
- Rarely or not at all (3)
